# The Kikuchi-Fujimoto Disease in Nigeria: A Case Report and Literature Review

**DOI:** 10.1155/2014/171029

**Published:** 2014-04-28

**Authors:** Akinsegun Akinbami, Mojeed Odesanya, Sunday Soyemi, Sarah John-Olabode, Adewumi Adediran, Olajumoke Oshinaike, Ebele Uche, Adedoyin Dosunmu, Akinola Dada, Olaitan Okunoye

**Affiliations:** ^1^Department of Haematology and Blood Transfusion, College of Medicine, Lagos State University, PMB 21266, Ikeja, Lagos, Lagos State, Nigeria; ^2^Oak Hospitals, 191 Lagos Road, Ikorodu, Lagos State, Nigeria; ^3^Department of Pathology and Forensic Medicine, College of Medicine, Lagos State University, PMB 21266, Ikeja, Lagos, Lagos State, Nigeria; ^4^Department of Haematology and Immunology, Ben Carson School of Medicine, Babcock University, Ilishan, Ogun State, Nigeria; ^5^Department of Haematology and Blood Transfusion, Faculty of Clinical Sciences, College of Medicine, University of Lagos, PMB 12003, Lagos State, Nigeria; ^6^Department of Medicine, College of Medicine, Lagos State University, PMB 21266, Ikeja, Lagos, Lagos State, Nigeria; ^7^Department of Medicine, University of Port Harcourt, PMB 5323, Choba, Port Harcourt 234, Rivers State, Nigeria

## Abstract

The Kikuchi-Fujimoto is a rare, self-limiting disease, which is characterized by regional lymphadenopathy. It occurs worldwide with a higher prevalence among Asians and women below the age of forty years. We present 41-year-old Nigerian woman who was investigated extensively for unilateral left cervical lymphadenopathy. She was eventually diagnosed as having the Kikuchi-Fujimoto disease and was managed conservatively thereafter. We describe a case report and review of literature for better awareness of the disease amongst medical practitioners and pathologists in Africa.

## 1. Background

The Kikuchi-Fujimoto disease (KFD) is also known by the names histiocytic necrotizing lymphadenitis, necrotizing granulomatous lymphadenitis, and Kikuchi's disease. KFD is a benign, extremely rare disease which is characterized by fever and regional (often tender) lymphadenopathy. This self-limiting disease, which often bears a clinicopathologic and diagnostic similarity to some malignancy or connective tissue diseases, was first described in 1972 in Japan, independently by Kikuchi [1] and by Fujimoto et al. [2]. The pathophysiology is not completely understood, but antigenic response to yet unidentified antigenic stimuli and autoimmune apoptotic processes are believed to play a pivotal role [3, 4]. KFD is sometimes a clinical challenge and is occasionally reported in the literature [5–12]. There has been no published report of cases of KFD in Nigeria as at time of this report. KFD has a worldwide distribution with a higher prevalence amongst Japanese and other Asians, with isolated cases reported in Europeans [13]. The disease most commonly occurs in people younger than forty years, with an age range of 19 months–75 years [14–17]. It was believed to have a female preponderance (m : f = 4 : 1), but recent studies have put the ratio closer to 1 : 1 [13, 17].

## 2. Case Report

A 41-year-old known hypertensive female lecturer who lives in Nigeria was managed for a 3-month history of left cervical lymph node enlargement, with partial response to two courses of antibiotics, but with recurrence afterwards. She neither smoked nor drank alcohol. A complete blood count showed neutropenia and a mildly reduced MCH of 26.3 pg. She had a heamatocrit of 36%, total leukocyte count of 4,600 cells/*μ*L, and absolute neutrophil and lymphocyte counts of 1,450 and 2610 cells/*μ*L, respectively. The erythrocyte sedimentation rate (ESR) was 22 mm/hour. Viral screens for HIV I and II, hepatitis B, and hepatitis C were negative. A neck ultrasound scan revealed bilateral small adenopathy, with the largest node, showing loss of fatty hilum in the left posterior triangle. She had a lymph node biopsy which suggested a lymphoproliferative disorder based on a loss of nodal architecture with infiltration of small lymphocytes and large areas of necrosis. A chest X-ray revealed bronchitic changes bilaterally, with mild cardiomegaly. The sputum for acid-fast bacilli was negative and the Mantoux test was nonreactive. A mammogram revealed no significant findings.

Immunohistochemistry revealed large zones of paracortical necrosis, with infiltration by CD68+ and myeloperoxidase (MPO)+ histiocytes full of apoptotic debris, surrounded by plasmacytoid dendritic cells (CD123+) and a background population of T lymphocytes with a decreased CD8+/CD4+ ratio. Neither was there evidence to suggest infection by EBV, fungi, or acid-fast bacilli, nor was there evidence indicative of a lymphoma. A clinical diagnosis of KFD was made. A separate histopathologic analysis at another laboratory confirmed the diagnosis. She had no significant symptoms and was observed. The cervical adenopathy has regressed completely and was symptom-free as at the time of this report, except for mild episodes of upper respiratory tract infection.

## 3. Discussion

KFD is an enigmatic disease of acute or subacute onset, evolving over a period of two to three weeks. It runs a self-limited course lasting one to four months. Typical presentation is cervical lymphadenopathy (56–98% of cases), often unilateral (88.5%), with tenderness (59%) and most frequently located in the posterior cervical triangle (88.5%) [13]. Lymph node sizes most commonly range from 0.5 to 4 cm. Generalised lymphadenopathy and extranodal involvement are very rare occurrences in KFD [18]. Our patient was a female, who presented with unilateral small cervical adenopathy in the left posterior triangle—the typical clinical features of KFD, although her age was slightly outside the peak range. She had no extranodal involvement.

Although there are no specific diagnostic tests available for KFD and diagnosis is generally made based on the histopathologic findings from an excision biopsy of affected lymph nodes. Laboratory investigations usually yield normal results, but a few patients have mild elevation of the ESR and mild anaemia and leucopenia [19]. Our patient had a mildly elevated ESR and a granulocytopenia. Leukopenia (especially granulocytopenia) occurs in 25% to 58% of patients, whereas leukocytosis is seen in 2% to 5% of cases [13]. Some authors have postulated that inhibitory factors in serum may be responsible for granulocytopenia in KFD [20].

The aetiopathogenesis of KFD remains incompletely understood. The clinical and histologic findings in KFD have prompted scientists to make postulations on associations with a variety of antigenic stimuli and associations with diseases such as systemic lupus erythematosus (SLE), lymphoma, tuberculosis, and Kawasaki's disease. A viral origin has been strongly suggested based on similarities in the typical clinical course, as well as the histopathologic features of KFD such as presence of necrosis in the T cell zones of the lymph nodes, infiltration by immunoblasts, expansion of the paracortex, and immunologic evidence of T cell predominance [20]. However, no viral particles have been isolated, and serologic tests for several viruses including the Epstein-Barr virus, cytomegalovirus, parvovirus B19, and herpes viruses have proved unconvincing [13, 19]. Viral screens in our case report were similarly negative. Other speculated aetiologic agents with inconclusive or negative results include* Yersinia enterocolitica* and* Toxoplasma gondii* [13].

The histopathologic features often utilized in the diagnosis of KFD include a distortion of nodal architecture, with paracortical necrosis, abundant karyorrhectic debris, and a large number of histiocytes (which are MPO+, CD68+, and express lysozyme) and plasmacytoid monocytes within the karyorrhectic foci. A large number of T cells are usually found, but granulocytes are usually absent. The presence of reactive atypia in the reactive immunoblastic component can be mistaken for a lymphoma [15]. The index case had large zones of paracortical necrosis and CD68+ and MPO+ histiocytes full of apoptotic debris, surrounded by plasmacytoid dendritic cells (CD123+) and a background population of T lymphocytes with a decreased CD8+/CD4+ ratio. Immunohistochemical studies have established CD8+ T cell as the predominant proliferating cell in affected lymph nodes [15, 21–24]. These cells have also been proposed to be both the inducers of the apoptotic process based on the findings of studies on the perforin and Fas pathways [25]. However CD8+ is also killed in the process [25]. This may account for the decreased CD8+/CD4+ ratio seen in our index case.

The clinical and histological features of KFD may mimic diseases like lymphomas, SLE, metastatic adenocarcinoma, Kawasaki's disease, mononucleosis, herpes infections, and tuberculosis and differentiation can be challenging [13, 25, 26]. It is speculated that KFD may be under diagnosis in clinical practice, as many mild forms with low grade fever and small cervical adenopathy without biopsies may have been wrongly diagnosed as viral infections [13]. KFD must be considered as a differential diagnosis in patients with fever, unilateral cervical adenopathy, and histological features showing large necrotic foci with evidence of apoptosis and infiltration by histiocytes. Recognition of KFD will avoid misdiagnosis. Supportive measures are the mainstay of therapy as no treatment is required in most cases of KFD.

The management of lymphadenopathy is a common medical problem worldwide. As KFD is rare, a high level of awareness is needed to suspect the diagnosis. Confirming the diagnosis early helps in the timely institution of proper management. This, in addition to a dearth of equipment and expertise for immunohistochemistry, may account for the absence of any reported diagnosis of KFD in our environment and indeed many African countries alike. We therefore recommend an increased awareness of the disease amongst clinicians and pathologists in Nigeria.
